# Association of semen cytokines with reactive oxygen species and histone transition abnormalities

**DOI:** 10.1007/s10815-016-0756-7

**Published:** 2016-06-30

**Authors:** Lu Jiang, Ting Zheng, Jun Huang, Jinhua Mo, Hua Zhou, Min Liu, Xingcheng Gao, Bolan Yu

**Affiliations:** 1 Key Laboratory of Reproduction and Genetics of Guangdong Higher Education Institutes, Third Affiliated Hospital of Guangzhou Medical University, Guangzhou, 510150 China; 2 Key Laboratory for Major Obstetric Diseases of Guangdong Province, Third Affiliated Hospital of Guangzhou Medical University, Guangzhou, 510150 China; 3 Rm 806, Teaching Building, No.63 Duobao Rd, Guangzhou, 510150 China

**Keywords:** Seminal cytokine, ROS, Semen quality, Histone-to-protamine transition

## Abstract

**Purpose:**

The aim of this study is to investigate the relationships among reactive oxygen species (ROS) elevation, histone transition, and seminal cytokine concentrations.

**Methods:**

Total levels of ROS in semen samples from 6560 men were measured. From this sample, 118 cases with high ROS and 106 controls were recruited. Basic semen parameters and histone-to-protamine ratios were analyzed, 400 semen cytokine and receptor alterations were assayed by protein chip, and finally 18 cytokines were validated in each sample using a Bio-Plex Cytokine assay.

**Results:**

The results showed that the seminal ROS concentration was associated with abnormalities in the sperm histone transition. Compared with controls, 93 cytokines had significant alterations in the high ROS cases, with 14 of them further verified in individual samples. The concentrations of CXCL5, CXCL16, CXCL8, IL-1b, IL-10, CSF3, CCL3, and TNF-α were significantly correlated with the histone transition ratio. In addition, IL-16 showed significantly different concentrations in controls, normal semen with high ROS levels, and abnormal semen with high ROS levels.

**Conclusions:**

Semen ROS are associated with abnormalities in sperm histone transition. CXCL5, CXCL8, IL-16, CCL8, CCL22, CCL20, CXCL16, IL-1B, IL-6, IL-7, IL-10, CSF3, CCL3, CCL4, and TNF-α all have elevated concentrations in semen with high ROS levels. These data might help to explain the mechanisms behind the increase in the levels of ROS and seminal cytokines and their relationship with defective spermatogenesis.

## Introduction

Male infertility has become a prevalent worldwide problem in recent decades. The incidence of infertility is approximately 15 %, with about 50 % due to male infertility [1]. Reactive oxygen species (ROS) constitute a potentially considerable environmental risk factor for infertility. Smoking, drinking, radiation, toxins, infection, and inflammation have all been correlated with elevated ROS in humans [2]. ROS play a potentially impactful role in male reproductive biology [3]: High levels of ROS in semen might damage cellular membranes and oxidize protective lipids and are therefore negatively correlated with many sperm function parameters [3–5]. In the clinic, elevated ROS levels are observed in patients with abnormal sperm motility and morphology, and oxidative stress plays a critical role in male factor infertility in those with high levels of ROS [6].

Cytokines are polypeptide regulatory factors that are involved in the immune response, cellular growth and differentiation, inflammation, and repair. In the male genital tract, cytokines are produced by the testes and regulate the proliferation of germ cells and differentiation of mesenchymal cells and play a part in steroid anabolism [7, 8]. Several cytokines in seminal plasma have been correlated with semen quality parameters such as sperm concentration, motility, viability, and morphology [9, 10]. For example, Naz et al. proposed that the concentration of interleukin (IL)-12 was positively correlated with the number and normal morphology of sperm [11], while Fraczek et al. determined that under in vitro conditions, IL-12 could bind to IL-18 and destroy the sperm plasma membrane and DNA integrity [12]. In addition, the levels of several cytokines (IL-6, IL-8, TNF-α, IL-10) are elevated in men with genital infections and could play roles in the immune defense of the male genital tract [13].

An increasing number of studies have reported in recent years that ROS and cytokines possess a complex and exquisite interplay. Although ROS can promote the expression and production of cytokines, some cytokines can modulate pro-oxidant and antioxidant systems and the generation of ROS [14–16]. Several studies have documented the association between cytokines and ROS. For instance, high levels of IL-6 and IL-8 promote the peroxidation process, affecting the function of sperm and causing infertility during inflammation of the male genital tract [14]. In infertile patients with varicoceles, increased levels of IL-6 and ROS might reduce the total antioxidant capacity [15]. In addition, men with chronic prostatitis and elevated levels of TNF-α in semen had increased physiologic concentrations of MDA (an oxidative stress product) in seminal plasma and damaged sperm function [16]. Therefore, seminal cytokines could be involved in various pathologic conditions connected to male infertility and interact with oxidative stress and cellular ROS, which might then lead to defective sperm function.

Histone-to-protamine transition is a key step in the establishment of epigenetic stability in spermatozoa, and the histone-to-protamine exchange rate in humans is around 85 % [17]. Abnormalities in histone-to-protamine transition in humans are associated with defective spermatogenesis and male infertility, and this process might be affected by oxidative stress in the testis [18]. Because it is not possible to directly measure the ROS levels in the human testis, an assay of seminal ROS could serve as an alternative. Several experiments have suggested a link between testicular oxidative stress and semen ROS levels. For instance, vitamin C has a protective effect in the testes of adult male rats, and seminal plasma ascorbic acid is negatively correlated with semen ROS signals [19, 20]. In the clinic, seminal ROS levels are significantly correlated with varicoceles, which can cause oxidative stress in the testis [21]. Therefore, the ROS detected in semen might reflect an imbalance between ROS generation and degradation in the male reproductive system, including the testis.

The aim of our present study was to investigate the relationships among high semen ROS, poor sperm quality, and seminal cytokine concentrations in infertile men. We screened the ROS concentrations of 6560 men and recruited 118 infertile males with high ROS as case samples and 106 normozoospermic males with low ROS as controls. We compared sperm function parameters, analyzed 400 semen cytokine alterations by protein chip technology, and further verified 18 cytokines in the two groups using Luminex xMAP technology with the Bio-Plex System.

## Materials and methods

### Subjects

This study was approved by the institutional review board of Guangzhou Medical University. All studies involving human subjects were conducted in accordance with Declaration of Helsinki guidelines. We obtained written informed consent from all participants and collected data regarding medical history and smoking status via a structured questionnaire. Study subjects were ethnic Han Chinese in southern China who had a semen examination in the Third Affiliated Hospital of Guangzhou Medical University from March 2014 to June 2015 for fertility assessment. Their smoking status was obtained by the survey and included the frequency of smoking (cigarettes/day) and years of smoking (years). After the clinical examination, the left part of the semen was used for ROS level analysis. In total, 14,278 semen samples were subjected to the initial ROS screening. Because most patients underwent at least two semen analyses in the clinic, 6560 men underwent a ROS measurement, with the initial results showing that 458 subjects had elevated ROS levels (ROS levels >40 relative light units [RLU]/s). We used 40 RLU/s as the critical value because it is the 95th percentile value for ROS levels among normal males [6].

Our study cases were then recruited from the 458 subjects based on the following inclusion criteria: (1) 20–40 years old, (2) consistent results from two or more semen tests, (3) semen samples collected by masturbation, and (4) no known diseases related to ROS elevation or no other conditions that might be related to ROS. Exclusion indications were (1) azoospermia, (2) hematospermia, (3) necrozoospermia (survival rate <58 %), (4) chromosomal or hormonal abnormalities, AZF deletions, or other known genetic diseases, (5) white cell contamination or polymorphonuclear granulocytes (PMNs) >1 × 10^6^/ml semen, and (6) diabetes, infections, or other diseases. Age- and smoking status-matched, normozoospermic controls were randomly picked. They were men with low ROS levels (ROS levels <40 RLU/s) who met all inclusion criteria and exclusion indications.

Finally, 118 cases and 106 controls were enrolled in this study, classified into the cases group and control group. Based on their sperm parameters, cases were further stratified into two subgroups: Case 1 (*n* = 81) included men with normal semen parameters and high ROS, and case 2 (*n* = 37) included men with abnormal semen parameters and high ROS.

### Semen collection and analysis

The semen samples were collected using the standard method according to the World Health Organization [22]. All semen specimens from patients and controls were collected by masturbation into sterile containers. Immediately after liquefaction, fresh sperm concentration and percent motility were assessed using a computer-assisted sperm analysis system (Hamilton Thorne, Beverly, MA). PMNs were enumerated by peroxidase staining under microscopy. For sperm morphology and vitality evaluations, seminal smears were stained and assessed under microscopy according to World Health Organization guidelines.

### Determination of ROS levels

The ROS levels in fresh semen were determined within 2 h of sample collection using 5-amino-2,3-dihydro-1,4-phthalazinedione (Luminol, Sigma) as the probe according to the literature [6]. First, 2 μl of 25 mM luminol (dissolved in dimethyl sulfoxide, Sigma) and 8 μl of 755 IU/ml HRP (260 IU/mg) were added to 200 μl of neat semen. ROS levels were determined by measuring chemiluminescence (BioTek Synergy H1, USA). The results are expressed as relative light units per second (RLU/s). After the parameters were measured, the sperm and seminal plasma were separated by centrifugation at 500×*g* and stored at −80 °C for further cytokine analysis.

### Aniline blue staining

The ratio of histone-to-protamine in sperm nuclei was measured using a Nucleoprotein Transition Test Kit (Huakang Company, Shenzhen, China). Briefly, 5 μl of prepared spermatozoa were spread onto a glass slide and allowed to dry. The smears were fixed in 3 % buffered glutaraldehyde in 0.2-M phosphate buffer (pH 7.2) for 30 min. The slides were then stained with 5 % aqueous aniline blue mixed with 4 % acetic acid (pH 3.5) for 5 min. Positive staining indicates abnormal histone-to-protamine transition. A total of 400 sperm cells were evaluated under microscopy, and the percentage of stained sperm heads was calculated.

### Protein chip analysis

Due to the large number of cases and controls, a mixture of samples was analyzed using a protein chip and differently expressed cytokines were validated in each sample using a Bio-Plex Cytokine assay (Bio-Rad). The seminal cytokines were initially analyzed by Quantibody Human Cytokine Antibody Array 9000 (RayBiotech, GA, US). All samples from controls and all samples from cases were mixed by equal volume to create a case mixture and control mixture. In total, 400 cytokines and receptors were analyzed in both control and case mixtures according to the manufacturer’s protocol. Briefly, slide chips with control and case mixtures were completely dried for 1–2 h and then closed and incubated overnight. After the overnight incubation, the spots were washed twice. After incubation with biotin-labeled antibody for 2 h at room temperature, each spot was washed with washing buffer and 70 μl of diluted fluorescent agent (streptavidin-biotin [Cy3 equivalent]) was added to each well. The device was then covered with aluminum foil to avoid exposure to light and incubated for 1 h at room temperature. Water droplets were completely removed by applying gentle suction. Fluorescence detection was analyzed using laser scanners (Axon GenePix, USA) using Cy3 or the green channel (at an excitation frequency of 532 nm). Experiments were conducted in triplicates for the final analysis.

### Assay of cytokine concentrations using the Bio-Plex System

Detection and quantification of cytokines in frozen thawed seminal plasma were performed using the Bio-Plex Cytokine assay (Bio-Rad). Prior to the experiment, we prepared solutions of standards, antibody dilutions, and streptavidin-PE. Experiments were conducted in accordance with the manufacturer’s instructions. First, 50-μl beads were added to pre-wet wells (for the filter plate only). After two washes, 50 μl of standards, blank, or samples were added to each well and incubated at room temperature with shaking at 850 rpm. After three washes with wash buffer, 25 μl of detection antibody was added and the wells were incubated for 30 min at room temperature with shaking at 850 rpm. After three washes, 50-μl streptavidin-PE was added and the wells were incubated for 10 min. Finally, the beads in each well were resuspended in 125 μl of assay buffer and shaken at 850 rpm for 30 s, and the entire plate was read on the Bio-Plex system.

### Statistical analyses

The independent sample *t* test was used to analyze numerical data in two groups. The Mann-Whitney test was used to analyze nonhomogenous variables. The Spearman correlation coefficient was used to examine the associations between ROS levels and semen parameters as well as ROS levels and cytokines. *P* values of <0.05 (two-sided probability) were interpreted as statistically significant. Statistical analyses were conducted using SPSS statistical software (version 13.0) (SPSS Inc., Chicago, IL).

## Results

### Age, smoking status, semen parameters, and ROS levels of study subjects

The summary statistics of the semen parameters analyzed in the different populations are presented in Table 1. There were 106 controls with low ROS levels and 118 cases with high ROS levels. The two groups had a similar average age, years of smoking, and smoking frequency, but the level of histone transition abnormalities was significantly different. Sperm parameters, including concentration, motility, vitality, and malformation rate, were significantly different between cases and controls because the cases group contained patients with both high ROS and abnormal semen parameters.

**Table 1:** Age, smoking status, semen parameters, and ROS levels of study subjects

Parameters	Controls	Cases
Subject number	106	118
Age (years)	32.42 ± 3.71	32.62 ± 4.04
Smoking years^a^	1.06 ± 1.37	1.32 ± 1.19
Smoking frequency	0.92 ± 1.18	1.09 ± 0.95
Sperm concentration (×10^6^ cells/ml)	76.27 ± 45.78	70.09 ± 56.69*
Semen volume (ml)	3.50 ± 1.39	3.00 ± 1.19**
Sperm progressive motility (%)	63.59 ± 9.65	47.75 ± 18.81**
Sperm immotility (%)	29.37 ± 10.68	46.66 ± 20.57**
Sperm vitality (%)	88.54 ± 3.54	81.36 ± 10.46**
Malformation rate (%)	95.11 ± 4.19	96.80 ± 3.04**
Histone transition abnormality^b^	9.79 ± 4.76	14.32 ± 8.65**
ROS range (RUL/s)	0–40	41–5627
Semen ROS (RUL/s/10^6^ sperm)	1.14 ± 2.12	260.87 ± 405.07

Controls: semen samples with low ROS; cases: semen samples with high ROS. Data are presented as mean ± SD unless otherwise noted

*P < .05 and **P < .01 compared with controls

^a^Information of smoking dose (packs per day) and years smoked was acquired from subjects

^b^The abnormality in histone transition was the percentage of sperm cells that had elevated histone-to-protamine ratios

### Correlations between ROS and sperm quality

ROS levels were correlated with most sperm quality parameters in the case subjects (Table 2), including sperm concentration, sperm vitality, sperm progressive motility, sperm immotility, and malformation rate. In addition, ROS levels were positively correlated with histone transition abnormalities.

**Table 2:** Correlations between ROS and sperm quality parameters

Parameters	*r*	*p*
Smoking frequency	0.113	0.091
Smoking duration	0.114	0.094
Age	−0.014	0.83
Sperm concentration	−0.429**	<0.01**
Semen volume	−0.147*	0.029
Sperm vitality	−0.412**	<0.01**
Sperm progressive motility	−0.558**	<0.01**
Sperm immotility	0.573**	<0.01**
Malformation rate	0.383	<0.01**
Histone transition abnormality	0.306**	<0.01**

**Correlation was significant at the 0.01 level (two-tailed)

### Protein chip analysis of cytokines and receptors in the study groups

Four hundred cytokines and receptors were analyzed in the case subjects and controls using protein chip analysis. Significantly differentially expressed cytokines were selected, as defined by a signal value greater than 200 and fold change (expression level in case subjects/expression level in controls) greater than 2 or less than 0.5 (Table 3). The final results revealed that 93 cytokines were differentially expressed between the case subjects and controls. Of these, the CXCL5, CCL22, CCL8, and TGFB1 levels were significantly higher in the cases, with fold changes greater than 10, and IL-5Ra and TACSTD1 were significantly lower in the case subjects, with fold changes less than 0.2.

**Table 3:** Protein-chip analysis of cytokines and receptors in cases and controls

Official symbol	Fold change^a^	Official symbol	Fold change^a^	Official symbol	Fold change^a^
CXCL5	15.3	LIF	3.4	SDC3	2.3
CCL22	14.8	FTL	3.3	GNLY	2.3
CCL8	11.5	EDA2R	3.3	GDF15	2.3
TGFBI	11.1	CXCL1	3.3	MCF R	2.3
CXCL6	9.4	NOTCH1	3.1	FRZB	2.3
CSF3	9.0	IL3	3.1	TNFRSF1B	2.2
IL1B	7.9	IL33	3.1	GDNF	2.2
S100A8	7.4	GAS1	3.1	IL16	2.2
IL7	6.5	F3	3.1	CD274	2.2
MICA	6.4	CCL20	3.0	RARRES2	2.2
MET	6.3	IL32	3.0	MUC16	2.2
IL6	6.2	C5a	3.0	IGFBP6	2.2
MMP9	6.1	PDCD1	2.9	CD80	2.2
CCL4	5.9	CCL7	2.9	LGALS1	2.2
C16orf77	5.7	CSF1	2.8	CRP	2.2
CCL3	5.7	FURIN	2.8	ADAM17	2.2
IL-18	4.9	CD97	2.7	BMP2	2.2
IL10	4.6	FGF19	2.6	HGF	2.2
MMP13	4.6	TNFRSF13B	2.6	PRSS8	2.1
IL8	4.4	PECAM1	2.5	CHL1	2.1
IL20	4.2	ROBO3	2.5	MME	2.1
ICAM1	4.2	CCL17	2.5	IL5	2.1
MMP10	4.0	IL17RA	2.5	CXCL9	2.0
CCL24	4.0	THBD	2.5	MMP8	2.0
RETN	3.9	CXCL16	2.4	TLR2	2.0
ADIPOQ	3.8	MMP1	2.4	PGLYRP1	2.0
CCL19	3.8	SIGLEC7	2.4	KIT	2.0
RGMB	3.7	CD200	2.3	LYVE1	0.3
SIGLEC5	3.6	CDH3	2.3	IL1F9	0.3
FAP	3.6	TNFRSF10D	2.3	IL5RA	0.2
WIF1	3.5	OLR1	2.3	TACSTD1	0.2

^a^Fold change = expression level in cases/expression level in controls

### Bio-Plex analysis of cytokines in study groups

To further confirm the protein chip assay data, cytokine concentrations were assayed in all 224 subjects using the Bio-Plex system (Table 4). The following 18 cytokines and chemokines were detected in this assay: CXCL5, CXCL6, CXCL8, IL-16, CCL8, CCL7, CCL22, CCL20, CCL19, CXCL16, IL-1B, IL-6, IL-7, IL-10, CSF3, CCL3, CCL4, and TNF-α. To distinguish between different subgroups in the case subjects, these samples were further stratified into two groups: Case group 1 comprised patients with normal semen parameters but high ROS, and the case group 2 comprised patients with abnormal semen parameters with high ROS. Most of the cytokines tested had significantly higher concentrations in the case groups than in the control subjects, including CXCL5, CXCL8, IL-16, CCL8, CCL20, IL-1B, IL-6, IL-7, IL-10, CSF3, CCL3, CCL4, and TNF-α. However, the differences in CXCL6, CCL7, CCL22, and CCL19 expression did not reach statistical significance. In addition, IL-16 showed significantly different concentrations between the control, case 1, and case 2 groups, respectively.

**Table 4:** Concentrations of cytokines in the different study groups

Cytokine	Controls (pg/ml)	Cases (pg/ml)	Case 1 (pg/ml)	Case 2 (pg/ml)	P1	P2	P3	P4
CXCL5	605.17	6107.71	4392.90	9870.78	<0.01**	<0.01**	<0.01**	0.20
CXCL6	8205.34	9026.83	9021.59	9038.19	0.855	0.951	0.759	0.855
CXCL8	505.63	2419.65	1919.96	3486.57	<0.01**	<0.01**	<0.01**	0.501
**IL16**	**54.33**	**345.66**	**186.09**	**686.37**	<0.01**	<0.01**	<0.01**	<0.05*
CCL8	65.06	255.24	319.75	113.68	<0.05*	<0.05*	<0.05*	0.546
CCL7	77.79	100.54	88.14	127.03	0.914	0.932	0.921	0.955
CCL22	669.89	1161.39	985.81	1536.28	0.088	0.128	0.206	0.815
CCL20	723.49	2605.39	2358.24	3133.09	<0.01**	<0.01**	<0.01**	0.600
CCL19	40.51	42.20	40.39	46.05	0.996	0.986	0.984	0.993
CXCL16	765.74	721.01	708.89	746.89	0.063	<0.05*	0.589	0.293
IL1B	1.63	30.88	25.15	44.03	<0.01**	<0.01**	<0.01**	0.256
IL6	16.90	103.44	66.22	188.82	<0.01**	<0.01**	<0.01**	0.062
IL7	863.28	690.68	728.74	603.38	<0.01**	<0.05*	<0.01**	0.082
IL10	2.81	14.52	11.42	21.65	<0.01**	<0.01**	<0.01**	0.580
CSF3	30.75	198.98	202.77	190.27	<0.01**	<0.01**	<0.01**	0.957
CCL3	2.06	36.02	21.93	68.35	<0.01**	<0.01**	<0.01**	0.339
CCL4	95.79	525.47	393.96	827.17	<0.01**	<0.01**	<0.01**	0.425
TNF-α	5.41	24.57	20.59	33.72	<0.01**	<0.01**	<0.01**	0.882

Controls, semen samples with low ROS. Case 1, normal semen parameters with high ROS. Case 2, abnormal semen parameters with high ROS. Cases: Case 1 + Case 2

*P1* control vs. cases, *P2* control vs. cases1, *P3* control vs. case 2, *P4* case 1 vs. case 2

*P < 0.05; **P < 0.01

### Correlations among cytokines, ROS, and sperm quality

Table 5 lists the correlations among cytokines, ROS, and sperm quality in all of our study subjects (cases plus controls). CCL3, CXCL5, CXCL8, CSF3, IL-10, IL-1B, TNF-α, and IL-6 were found to be significantly correlated with the histone transition ratio in seminal plasma. Sperm concentration was negatively correlated with IL-6, sperm vitality was negatively correlated with CXCL5, CXCL8, IL-1b, IL-6, IL-10, TNF-α, CSF3, CCL3, IL-16, CCL20, and CCL8, and sperm progressive motility was also negatively correlated with CXCL5, CXCL8, IL-1b, IL-6, IL-10, TNF-α, CSF3, CCL3, IL-16, CCL20, and CCL8. Moreover, ROS concentrations were positively correlated with the concentrations of CXCL5, CXCL8, IL-1b, IL-6, IL-10, TNF-α, CSF3, CCL3, IL-7, IL-16, CCL20, and CCL8.

**Table 5:** Correlations among cytokines, ROS, and sperm quality in all subjects

Cytokines	CXCL5/ENA-78		CXCL8/IL-8		IL1b		IL6	
	*r*	*p*	*r*	*p*	*r*	*p*	*r*	*p*
Sperm concentration	−0.045	0.519	−0.020	0.768	−0.126	0.070	−0.174	<0.05*
Sperm vitality	−0.294	<0.01**	−0.323	<0.01**	−0.319	<0.01**	−0.334	<0.01**
Sperm progressive motility	−0.226	<0.01**	−0.360	<0.01**	−0.384	<0.01**	−0.403	<0.01**
Malformation rate	0.070	0.317	0.134	0.052	0.145	<0.05*	0.269	<0.01**
Histone transition abnormality	0.319	<0.01**	0.230	<0.01**	0.204	<0.05*	0.194	<0.05*
ROS	0.541	<0.01**	0.680	<0.01**	0.716	<0.01**	0.659	<0.01**
Cytokines	IL10		TNF-α		CSF3/G-CSF		CCL3/MIP-1a	
	*r*	*p*	*r*	*p*	*r*	*p*	*r*	*p*
Sperm concentration	−0.108	0.120	−0.081	0.247	−0.122	0.080	−0.129	0.063
Sperm vitality	−0.303	<0.01**	−0.239	<0.01**	−0.299	<0.01**	−0.319	<0.01**
Sperm progressive motility	−0.338	<0.01**	−0.378	<0.01**	−0.371	<0.01**	−0.397	<0.01**
Malformation rate	0.210	<0.01**	0.208	<0.01**	0.201	<0.01**	0.208	<0.01**
Histone transition abnormality	0.207	<0.05*	0.277	<0.01**	0.214	<0.05*	0.224	<0.05*
ROS	0.676	<0.01**	0.667	<0.01**	0.704	<0.01**	0.752	<0.01**
Cytokines	IL7		IL16		CCL20/MIP-3a		CCL8/MCP-2	
	*r*	*p*	*r*	*p*	*r*	*p*	*r*	*p*
Sperm concentration	0.100	0.149	−0.107	0.122	−0.032	0.639	−0.011	0.873
Sperm vitality	0.071	0.309	−0.320	<0.01**	−0.215	<0.01**	−0.153	<0.05*
Sperm progressive motility	0.153	<0.05*	−0.315	<0.01**	−0.178	<0.01**	−0.111	0.108
Malformation rate	0.006	0.935	0.185	<0.01**	0.059	0.393	0.088	0.207
Histone transition abnormality	−0.051	0.564	0.141	0.110	0.058	0.511	−0.101	0.257
ROS	−0.233	<0.01**	0.647	<0.01**	0.473	<0.01**	0.155	<0.05*

Analyzed by Spearman’s correlation analysis, *P < 0.05, **P < 0.01

## Discussion

The present study is the first to report that semen ROS is associated with abnormalities in sperm histone transition (Table 2). Because protamines in mature sperm can change DNA neutrality, inhibit RNA synthesis, and prevent sperm gene expression in seminal plasma [18, 23, 24], an abnormal histone-to-protamine transition may interfere with the stability of sperm DNA, disrupt normal depolymerization in sperm nuclei, and eventually lead to decreased sperm fertilizing potential and embryonic development [25, 26]. In this way, high ROS levels may, by interfering with sperm epigenetic stability, be one of the risk factors for unexplained miscarriages and embryo development failure.

The detailed mechanisms involved in ROS induction of histone transition abnormalities have not yet been reported, but recent studies have found that ROS could lead to alterations in sperm DNA methylation [27]. A molecular study suggested that oxidative stress and ROS can impair the process of DNA methylation by inhibiting substrate synthesis or decreasing the activity of epigenetic enzymes such as DNMT and HDAC [28–31]. It is unclear if ROS can impair the histone-to-protamine transition in a similar manner and further investigation is thus warranted.

The literature shows that cytokines related to male genital tract infections may promote the generation of ROS [32, 33]. Several previous studies also showed that interleukins such as IL-6 and IL-8 and the cytokine TNF-α play an important role in the inflammatory process [15, 34]. In our study, the concentrations of many immune cytokines were elevated in the case groups with high levels of ROS but without infections or other diseases (Tables 3 and 4). For instance, IL-16 is a CD4 chemoattractant, CXCL8 (IL-8) is a PMN chemoattractant, IL-1B is a leukocyte activator that increases endothelium adhesion, IL-6 is a hematopoiesis factor related to the differentiation and inflammation of T and B cells, IL-7 is a pre-/pro-b cell proliferation and pro-inflammatory cytokine, IL-10 is a T and B cell activator that inhibits IFN-r, TNF-b, and IL-2, CSF3 (G-CSF) stimulates the growth and differentiation of granulocytes, TNF-α is a mediator of inflammatory reactions, and CXCL5 (ENA-78), CCL8 (MCP-2), CCL2 (MIP-3a), CCL (MIP-1a), and CCL4 (MIP-1b) are all chemokines that stimulate leukocyte movement. Therefore, elevated levels of these cytokines in patients with high ROS levels suggest that excessive ROS might be associated with the immune response, even though no immune diseases were detected.

Validation of some cytokine alterations in each subject showed that certain cytokines found to be altered in the protein chip assay were also changed at the individual level. Cytokines such as CXCL5 (ENA-78), CXCL8 (IL-8), IL-16, IL-1b, IL-6, IL-10, CCL3 (MIP-1a), and CCL4 (MIP-1b) exhibited increased levels in semen with high ROS compared with controls, suggesting that their concentrations are tightly linked to seminal ROS levels (Table 4). One cytokine, IL-16, showed significantly different concentrations among controls, normal semen with high ROS, and abnormal semen with high ROS (Table 4).

Further analysis revealed that although the aforementioned cytokines all appeared to be associated with semen ROS and sperm quality, they could potentially be classified into three types of cytokines (Table 5). The first of these types, which included CXCL5 (ENA-78), CXCL8 (IL-8), IL-1b, IL-6, IL-10, TNF-α, CSF3 (G-CSF), and CCL3 (MIP-1a), was associated with all semen quality parameters including concentration, vitality, motility, histone transition abnormalities, and semen ROS levels. The second type, including IL-7, IL-16, and CCL20 (MIP-3a), was associated with common semen parameters and semen ROS, but not histone transition abnormalities. The third type of cytokine, including CCL8 (MCP-2), was associated with only semen vitality and ROS. We speculated that this classification reflects the degree of association of cytokines with spermatogenesis. If the cytokines are associated with several pathways in spermatogenesis, we concluded that ROS may be linked with additional semen parameters. Based upon this possibility, cytokines such as TNF-α, CSF3 (G-CSF), and CCL3 (MIP-1a) are thus more likely to be closely associated with defective spermatogenesis.

Our current data also revealed that certain cytokines were significantly correlated with the histone-to-protamine ratio (Table 5). The concentrations of CXCL5, CXCL16, and TNF-α were found to be significantly correlated with the histone transition ratio, suggesting that cytokines are involved in mechanisms of altered protamine expression and excessive ROS and that they exist in a complex regulatory interplay during histone transition. This linkage might exist because cytokines are directly involved in the histone transition process or because histone transition is highly associated with sperm quality. However, we do not yet have enough details on this linkage.

In conclusion, we have found in our present study that the semen ROS levels are associated with abnormalities in the sperm histone transition pathway. CXCL5, CXCL8, IL-16, CCL8, CCL22, CCL20, CXCL16, IL-1B, IL-6, IL-7, IL-10, CSF3, CCL3, CCL4, and TNF-α all have elevated concentrations in semen with high ROS levels. In addition, IL-16 shows significantly different concentrations among normal semen with low ROS levels, normal semen with high ROS levels, and abnormal semen with high ROS levels. Therefore, our current findings provide further insights into the causes of high ROS and the underlying mechanisms.
